# *Ulva pertusa,* a Marine Green Alga, Attenuates DNBS-Induced Colitis Damage via NF-κB/Nrf2/SIRT1 Signaling Pathways

**DOI:** 10.3390/jcm11154301

**Published:** 2022-07-25

**Authors:** Alessio Ardizzone, Alessia Filippone, Deborah Mannino, Sarah Adriana Scuderi, Giovanna Casili, Marika Lanza, Laura Cucinotta, Michela Campolo, Emanuela Esposito

**Affiliations:** Department of Chemical, Biological, Pharmaceutical and Environmental Science, University of Messina, Viale Ferdinando Stagno D’Alcontres, 31-98166 Messina, Italy; aleardizzone@unime.it (A.A.); alessia.filippone@unime.it (A.F.); debmannino@unime.it (D.M.); sarahadriana.scuderi@unime.it (S.A.S.); gcasili@unime.it (G.C.); mlanza@unime.it (M.L.); laura.cucinotta@unime.it (L.C.); eesposito@unime.it (E.E.)

**Keywords:** inflammatory bowel disease (IBD), *Ulva pertusa*, anti-inflammatory, antioxidant, SIRT1/Nrf2 signaling, apoptosis, NF-κB

## Abstract

Inflammatory bowel diseases (IBD) including Crohn’s disease (CD) and ulcerative colitis (UC) represent gastrointestinal (GI) disorders associated with varied responses to microbial and environmental agents. Natural compounds have been suggested as a valid approach to the management of various GI diseases, particularly the green alga *Ulva pertusa*, belonging to the *Ulvaceae* family, which showed powerful biological properties. Here, we aimed to evaluate the effect and the mechanism of *Ulva pertusa* treatments in a murine model of DNBS-induced colitis. Colitis was induced by DNBS intrarectal installation (4 mg in 100 μL of 50% ethanol), while *Ulva pertusa* treatments (doses of 10, 50 and 100 mg/kg) were administered orally daily. *Ulva pertusa*, at the higher doses of 50 and 100 mg/kg, significantly reduced tissue damage DNBS-induced and the consequent inflammatory cascade via NF-κB inhibition. Furthermore, we demonstrated, for the first time, *Ulva pertusa* action on the SIRT1/Nrf2 axis, enhancing antioxidant response and the modulation of the apoptosis pathway colitis-induced, regulating the expression of p53, Bax, Bcl-2, and Caspases. Taken together, *Ulva pertusa* could be considered a valid approach for counteracting and blocking the progression of IBDs through modulation of the NF-κB/SIRT1/Nrf2 axis.

## 1. Introduction

Inflammatory bowel diseases (IBD) refer to a group of chronic disorders that affect the gastrointestinal (GI) tract with relapse-remitting symptomatology [1]. Chronic disorders include Crohn’s disease (CD) and ulcerative colitis (UC), which are the most common and debilitating worldwide conditions [2], usually characterized by weight loss, diarrhea, rectal bleeding, abdominal pain, and fatigue, by impacting patients’ lives, social relationships, and daily activities [3,4].

Although the etiology of IBD is unknown, several articles [5,6] have highlighted the prominent role of inflammatory pathways, and oxidative/nitrosative stress in the context of IBD development, by providing a new perspective on the discovery of efficacious treatments and management of these disorders. In this regard, a deep understanding of antioxidant pathways involved in IBD could result in a useful comprehension of efficacious approaches [7]. The interaction between NF-κB pathway activation and the accumulation of ROS and RNS translates into intestinal inflammation and oxidative stress by modulation of NF-κB/Nrf-2 and silent information regulator 2-related protein- 1 (SIRT1). These multifunctional factors, involved in the regulation of apoptosis, aging, and homeostasis signaling pathways, have been reported to possess roles in counteracting inflammatory common conditions in IBD [7]. From that perspective, natural products’ use in counteracting inflammatory diseases of GI has gained attention in order to understand their great potential and benefits for human health. In particular, the green alga *Ulva pertusa* is an important food source in many parts of the world, being nutrient-rich in dietary fiber, polysaccharides, protein, lipids, vitamins, and minerals [8,9].

Thanks to its biological properties, such as antihyperlipidemic and immunomodulatory, and especially its powerful antioxidant activity, *Ulva pertusa* is widely used in traditional Chinese medicine as a medication for many human diseases [8], thus representing a promising natural compound for functional food and drug development [9]. Despite previous reports [10,11] that have highlighted the activity of *Ulva* extracts in providing intestinal relief, the knowledge of the multiple molecular pathways with which it acts is still poor.

Therefore, in the present study, we investigated the properties of *Ulva pertusa* extract, composed of a significant portion of polysaccharides, proteins, and a lipid fraction (See Supplementary File). In recent years, polysaccharides, such as rhamnose, obtained from natural resources have been reported to offer benefits in terms of safety, high therapeutic efficacy, non-toxicity, low cost, and biocompatibility [12]. These assumptions were also confirmed by in vivo studies where the anti-inflammatory effects of polysaccharides were strongly highlighted [13]. Similarly, amino acid administration, such as serine and glutamic acid, exhibited a reduction in colonic inflammation and T-cell infiltration into the lamina propria [14,15]. *Ulva pertusa* extract also consists of a lipid part, with a good percentage of polyunsaturated fatty acids (PUFA). Relatively, it is known how ω-3 and ω-6 PUFA acts upon intracellular signaling pathways, transcription factor activity, and gene expression, resulting in efficiency in the management of the colitis [16,17].

On this basis, this study aimed to go deeper into the pharmacological effect of *Ulva pertusa* extract in a mouse model of DNBS-induced colitis.

## 2. Materials and Methods

### 2.1. Materials

Unless otherwise stated, all compounds were obtained from Sigma–Aldrich (Milan, Italy). All chemicals were of the highest commercial grade available. All stock solutions were prepared in non-pyrogenic saline (0.9% NaCl; Baxter, Liverpool, UK). *Ulva pertusa* extract was kindly gifted by the Chemical Department of the University of Messina.

### 2.2. Animals

The study was performed using male CD1 mice (Envigo, Milan, Italy) at 4 weeks of age with a weight of 20–25 g. Mice were housed in a controlled environment (22 ± 2 °C, 55 ± 15% relative humidity, 12 h light/dark cycle), with food and water ad libitum. Before starting the study, the animals were maintained in a quarantine area for one week. During this stage, they were observed daily. Moreover, a numbered tag placed through the edge of the right ear identified the animals selected for the study. The animal study was performed in accordance with Italian regulations on the use of animals (D.M.116192) and Directive legislation (EU) (2010/63/EU) amended by Regulation (EU) 2019/1010.

### 2.3. Induction of Experimental Colitis

Colitis was induced by a single intrarectal administration on day 1 with a very low dose of DNBS (4 mg per mouse) as previously described by Casili et al. [18]. In detail, mice were anesthetized by Enflurane; subsequently, 2,4,6-dinitrobenzene sulphonic acid (DNBS; 4 mg in 100 μL of 50% ethanol) was injected into the rectum through a catheter inserted 4.5 cm proximally to the anus. In sham groups, vehicle alone was administered instead of DNBS.

After intrarectal administration, the animals were kept for 15 min in a Trendelenburg position to avoid reflux. After colitis and sham-colitis induction, the animals were observed and weighed daily.

At the end of the experiment, animals were sacrificed, and the colon was removed by surgical procedure and processed for histological examinations and biochemical analyses.

### 2.4. Experimental Groups

Mice were randomly divided into the following groups as summarized in Table 1.

*Ulva pertusa* extract water-soluble compound was administered orally after dissolution in saline [19,20]. The used doses were chosen based on a dose-response pilot experiment. Significant changes were neither reported nor observed in control mice treated with *Ulva* (Sham + *Ulva pertusa* 10 mg/kg, 50 mg/kg and 100 mg/kg groups) compared to the Sham + vehicle group; thus, no data of these groups were shown.

### 2.5. Histological Evaluation

Histological analyses were performed as previously described [21,22].

Briefly, after the sacrifice of the animals, colon tissues were immediately fixed in 10% (*w*/*v*) PBS-buffered formaldehyde solution at 25 °C for 24 h. After a dehydration process through a scale of increasing concentrations of alcohols and xylene, tissues were included in paraffin (Bio-Optica, Milan, Italy) and subsequently cut under the microtome in order to obtain 5 μm thick sections. For morphological analyses, slides were stained with Hematoxylin/Eosin (H&E, Bio-Optica, Milan, Italy) so as to assess histological alterations, edema, and neutrophilic infiltration. As reported by Colombo et al. [23], the following morphologic criteria were considered: score 0, no histological damage; score 1, focal epithelial edema and necrosis; score 2, diffuse swelling and necrosis of the villi; score 3, presence of neutrophil infiltrate in the submucosa; score 4, necrosis with neutrophil infiltrate; and score 5, massive neutrophil infiltrate and hemorrhage. All sections were examined using an Axiovision Zeiss (Milan, Italy) microscope in a blinded manner. The results of the histological examinations were displayed at 10× magnification (100 µm scale bar) and 20× magnification (50 µm scale bar).

### 2.6. Toluidine Blue Staining

To assess the number of mast cells in colon tissues, the sections were stained with toluidine blue as previously described [24]. Briefly, after the sacrifice of the animals, colon tissues were immediately fixed in 10% (*w*/*v*) PBS-buffered formaldehyde solution at 25 °C for 24 h. After a dehydration process through a scale of increasing concentrations of alcohols and xylene, tissues were included in paraffin (Bio-Optica, Milan, Italy) and subsequently cut under the microtome in order to obtain 5 μm thick sections. Sections were deparaffinized in xylene and dehydrated through a graded series of alcohols; then, the sections were placed in water, stained with toluidine blue, and blotted carefully. Sections were placed in absolute alcohol, cleared in xylene, and mounted on a glass slide using Eukitt (Bio-Optica, Milan, Italy).

Mast cells count was performed on each slide using an Axiovision Zeiss microscope (Milan, Italy). For toluidine blue staining, 40× magnification (20 µm scale bar) was shown.

### 2.7. Immunohistochemical Analysis of iNOS, COX-2 and Nitrotyrosine

Immunohistochemical localization was made, as previously described [25,26].

Colon samples were immediately fixed in 10% (*w*/*v*) PBS-buffered formaldehyde solution at 25 °C for 24 h. After a dehydration process through a scale of increasing concentrations of alcohols and xylene, tissues were included in paraffin (Bio-Optica, Milan, Italy) and subsequently cut with microtome into 7 µm slices and, after deparaffinization, endogenous peroxidase was quenched with 0.3% (*v*/*v*) hydrogen peroxide in 60% (*v*/*v*) methanol for 30 min. Slides were permeabilized with 0.1% (*w*/*v*) Triton X-100 in PBS for 20 min. Non-specific adsorption was decreased by incubating the section in 2% (*v*/*v*) normal goat serum in PBS for 20 min. Endogenous avidin or biotin binding sites were blocked by sequential incubation for 15 min with avidin and biotin (Vector Laboratories, Burlingame, CA, USA), respectively.

Thereafter, slices were incubated at room temperature overnight with one of the following primary antibodies: anti-iNOS (BD Biosciences #610432, 1:100 in PBS, *v*/*v*), anti-COX-2 (Santa Cruz Biotechnology sc-376861, 1:100 in PBS, *v*/*v*), or anti-Nitrotyrosine (Cayman #189542, 1:100 in PBS, *v*/*v*). At the end of the overnight incubation with the primary antibody, slides were washed with PBS and incubated with a secondary antibody (Santa Cruz Biotechnology, Dallas, TX, USA) for 1 h. The reaction was revealed by a chromogenic substrate (brown DAB), and counterstaining with Nuclear Fast Red. To prove the binding specificity for different antibodies, some sections were also incubated with only primary antibody or secondary antibody; no positive staining was observed in these sections. All stained sections were observed and analyzed in a blinded manner. For immunohistochemistry, 20× (50 µm scale bar) and 40× (20 µm scale bar) were shown.

### 2.8. Myeloperoxidase (MPO) Activity

MPO activity, an index of neutrophil infiltration, was estimated as previously described [24,27]. The rate of change in absorbance was calculated spectrophotometrically at the wavelength of 650 nm. MPO activity was expressed in U per gram weight of wet tissue and was quantified as the quantity of enzyme degrading 1 μmol peroxide/min at 37 °C.

### 2.9. Malondialdehyde (MDA) Assay

MDA level is a useful indicator of lipid peroxidation. MDA assay in the colon tissues was determined as previously described [28,29].

### 2.10. Western Blot Analysis of iNOS, COX-2, IκB-α, NF-κB, MnSOD, Nrf2, HO-1, SIRT1, p53, Bcl-2, Bax, p-IκB-α and p-NF-κB

Western blot analysis was performed as previously described [25,30,31].

Briefly, after protein extraction from colon tissues, lysates were used for the detection of iNOS, COX-2, IκB-α, MnSOD, HO-1, and SIRT1 at the cytosolic level and the detection of NF-κB and Nrf2 at the nuclear level. Following SDS-PAGE, proteins are transferred to the PVDF membrane.

Membranes were incubated at 4 °C overnight with each of the following primary antibodies: anti-iNOS (1:500; BD Biosciences #610432), anti-COX-2 (1:500; Santa Cruz Biotechnology sc-376861, Dallas, TX, USA), anti-IκB-α (1:500; Santa Cruz Biotechnology sc-1643, Dallas, TX, USA), anti-NF-κB (1:500; Santa Cruz Biotechnology sc-8008, Dallas, TX, USA), anti-MnSOD (1:500; Millipore #06-984), anti-Nrf2 (1:500; Santa Cruz Biotechnology sc-365949, Dallas, TX, USA), anti-HO-1 (1:500; Santa Cruz Biotechnology sc-136960, Dallas, TX, USA), anti-p53 (Cell Signaling Technology, #9282), anti-Bax (Cell Signaling Technology, #2772), anti-Bcl-2 (Cell Signaling Technology, #2876), anti-SIRT1 (1:500; Santa Cruz Biotechnology sc-74465, Dallas, TX, USA), p-IκB-α (1:500; Santa Cruz Biotechnology sc-8404, Dallas, TX, USA), and p-NF-κB (1:500; Santa Cruz Biotechnology sc-166748, Dallas, TX, USA) dissolved in a PMT solution containing: 1× phosphate buffer saline (PBS), 5% w/v nonfat dried milk powder, and 0.1% Tween-20. Later, membranes were washed and incubated with secondary antibody (1:1000, Jackson ImmunoResearch, West Grove, PA, USA) for 1 h at room temperature. To verify that the samples used contained a uniform concentration of protein lysates, they were incubated in the same way, with primary anti-β-actin antibody (1:500; sc-47778; Santa Cruz Biotechnology, Dallas, TX, USA) or LAMIN A/C (1:500; sc-47778; Santa Cruz Biotechnology, Dallas, TX, USA). Signals were exposed with chemiluminescence (ECL) detection system reagent according to the manufacturer’s instructions (Thermo, Waltham, MA, USA). The relative expression of the protein bands was quantified by densitometry and standardized to β-actin or LAMIN A/C levels as an internal control.

### 2.11. Enzyme-Linked Immunosorbent Assay (ELISA) Kits

ELISA kits were used to detect the levels of IL-5 (#BMS610), IL-13 (#BMS6015), IL-9 (#88-8092), IL-4 (#BMS613), GSH (#MBS266871), CAT (#MBS160589), SOD (#MBS034842), caspase-3 (#MBS849298-100), caspase-9 (#LS-F21324), and caspase-8 (#LS-F32785) in serum or colon tissues of mice as previously described [32,33], and according to the manufacturer’s protocols.

### 2.12. Nitric Oxide (NO) Measurements

NO assay was performed as previously described [26]; the absorbance was read spectrophotometrically at 540 nm.

### 2.13. Statical Analysis

Experimental data are expressed as mean ± standard error (SD) of N observations, in which N represents the number of animals studied. In the experiments involving histological evaluations, images are representative of at least three independent experiments. Data analysis was performed with one-way and two-way ANOVA followed by a Bonferroni post-hoc test for multiple comparisons. Only a *p*-value less than 0.05 was considered significant.

## 3. Results

### 3.1. Ulva pertusa Administration Reduced Weight Loss and Histological Damage following DNBS-Induced Colitis

**Figure 1:**
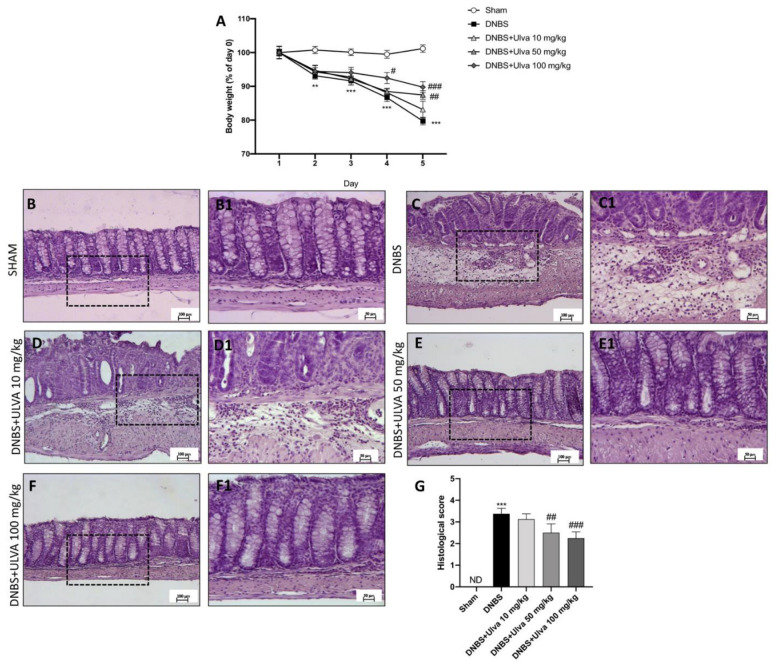
Effects of *Ulva pertusa* on body weight and histological damage. A decrease in body weight was observed in mice of the DNBS group compared to sham animals (**A**). Only *Ulva* at doses of 50 mg/kg and 100 mg/kg significantly reduced weight loss (**A**). Four days after DNBS intrarectal injection mice showed a marked alteration of tissue morphology (**C**), 20× magnification (**C1**), score (**G**), compared to the control group (**B**), 20× magnification (**B1**), score (**G**). Treatment with *Ulva* at the highest doses restored colon architecture (**E**), 20× magnification (**E1**) and (**F**), 20× magnification (**F1**), score (**G**), unlike treatment with the lowest dose which did not exert beneficial effects (**D**), 20× magnification (**D1**), score (**G**). Data are representative of at least three independent experiments. Values are means ± SD. One-way ANOVA test followed by Bonferroni. One-way and two-way ANOVA test. ** 0.01 vs. Sham; *** *p* < 0.001 vs. Sham; # *p* < 0.05 vs. DNBS; ## *p* < 0.01 vs. DNBS; ### *p* < 0.001 vs. DNBS; ND: not detectable.

The severity of ulcerative colitis is closely related to nutrient malabsorption which, associated with gastric symptoms onset, exposes individuals to severe body weight loss [34] and deficiency of mineral ions homeostasis [35]. According to previous studies, DNBS instillation induced significant weight loss in mice, from early days, compared to the control mice (Figure 1A). However, treatment with *Ulva* at a dose of 50 mg/kg, and even more effectively at a dose of 100 mg/kg, reduced weight loss due to DNBS (Figure 1A). No significant changes were observed by *Ulva* treatment at the lower dose of 10 mg/kg compared to the DNBS group (Figure 1A).

Moreover, histopathological changes due to DNBS intrarectal instillation revealed severe inflammation extent, loss of crypt architecture, edema, and the extent of infiltration with inflammatory cells damage extending in the submucosa layers of colon tissue (Figure 1(C1), see histological score Figure 1G) compared to the control mice in which the colon tissue structure has been kept regular (Figure 1(B1), see histological score Figure 1G).

The lower dose of *Ulva* (10 mg/kg) did not show significant attenuation of tissue damage (Figure 1(D1), see histological score Figure 1G) instead of the significant amelioration of tissue architecture and reduction of inflammation shown by *Ulva* treatments at the higher doses of 50 mg/kg (Figure 1(E1), see histological score Figure 1G) and 100 mg/kg (Figure 1(F1), see histological score Figure 1G).

### 3.2. Ulva Treatments Reduced Mast Cells Degranulation/Number and MPO Activity

**Figure 2:**
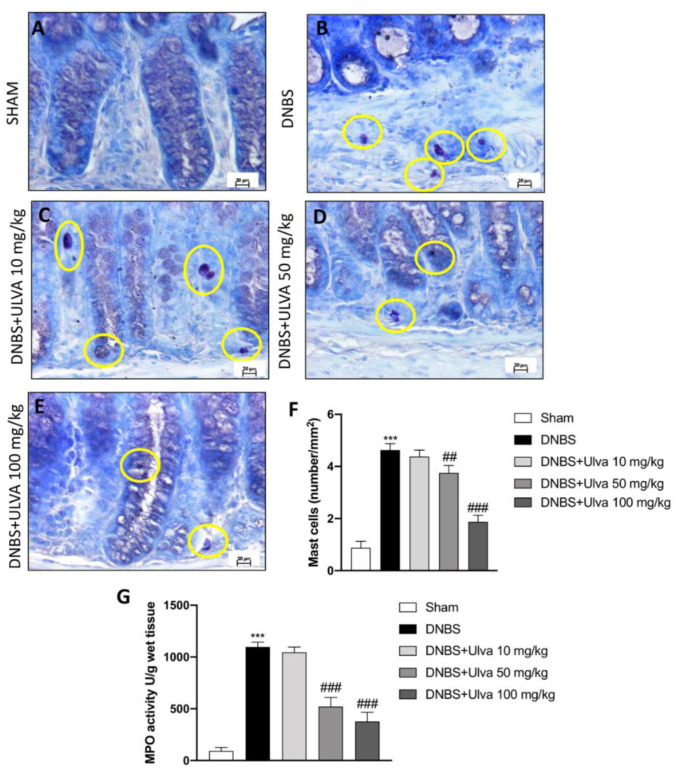
Effect of *Ulva pertusa* on mast cells in colon tissues after DNBS-induced colitis. A high number of mast cell (**B**,**F**) and neutrophil infiltration (**G**) was found in mice with DNBS-induced colitis, compared to control animals (**A**,**F**,**G**). *Ulva pertusa* 10 mg/kg was ineffective in reducing mast cell count and MPO activity (**C**,**F**,**G**). While a decreased number of mast cells and MPO activity was identified in mice treated with *Ulva* at doses of 50 mg/kg (**D**,**F**,**G**) and 100 mg/kg (**E**–**G**). Mast cells were highlighted by the yellow circles. Data are representative of at least three independent experiments. Values are means ± SD. One-way ANOVA test followed by Bonferroni. *** *p* < 0.001 vs. Sham; ## *p* < 0.01 vs. DNBS; ### *p* < 0.001 vs. DNBS.

A growing literature reveals increased mast cell degranulation and number in the human ulcerative colitis (UC) [36]. We detected an increased number of mast cells as well as their degranulation in colon tissue of DNBS-injected mice (Figure 2B, mast cell counts Figure 2F) compared to the control animals (Figure 2A, mast cells count Figure 2F). By contrast, a lower number of mast cells was detected in colon tissues from DNBS animals treated with *Ulva* 50 mg/kg (Figure 2D, mast cells count Figure 2F) and especially with *Ulva* 100 mg/kg (Figure 2E, mast cells count Figure 2F) despite no significant changes showed by *Ulva* treatment at the lower dose of 10 mg/kg (Figure 2C, mast cells count Figure 2F). Moreover, since neutrophil-myeloperoxidase (MPO) is an abundant granule enzyme that catalyzes the production of ROS in UC, we found a notable increase in MPO activity in DNBS-induced colitis mice when compared to the control mice (Figure 2G). The administration of *Ulva* at both doses of 50 mg/kg and 100 mg/kg was found to be efficient in reducing MPO activity; no significant reduction of MPO activity was shown by *Ulva* treatment at the lower dose of 10 mg/kg, thus, we did not consider it for further analysis.

### 3.3. Ulva pertusa Treatments Attenuated Inflammation Driven by the NF-κB Pathway and Modulated Pro-Inflammatory Interleukins Production

The inflammatory response is considered an important indicator of the inflammatory state of colon disease associated with increased transcription of the nuclear factor NF-κB [37].

**Figure 3:**
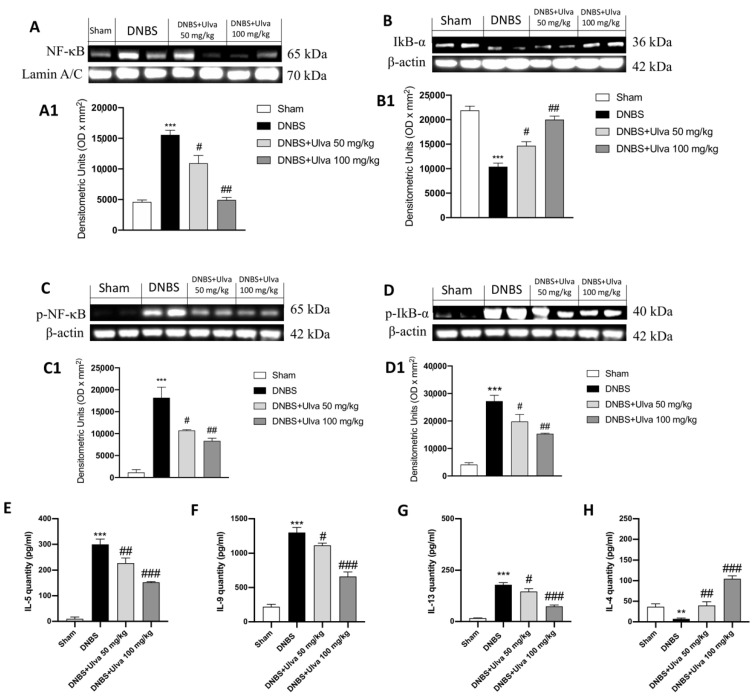
Effect of *Ulva pertusa* on NF-κB pathway and interleukin’s production. IκB-α basal levels were identified in sham mice, differently an increase in the degradation of IκB-α was found in the DNBS-vehicle group (**A**), densitometric analysis (**A1**). Against, NF-κB expression was significantly increased in the DNBS group compared to the sham group (**B**), densitometric analysis (**B1**). However, *Ulva pertusa* 50 mg/kg and 100 mg/kg restored IκB-α expression (**A**), densitometric analysis **A1** and decreased NF-κB one (**B**), densitometric analysis **B1**. These results were also confirmed by the evaluation of phosphorylated proteins (**C**), densitometric analysis (**C1**) and (**D**), densitometric analysis (**D1**). In serum, a substantial increase in the levels of interleukins -5, -9, and -13 was detected in the animals of the DNBS group compared to the Sham group (**E**–**G**). Treatment with *Ulva pertusa* reduced this expression in a dose-dependent manner (**E**–**G**). In contrast, IL-4 was decreased in DNBS mice compared to the control group (**H**); treatment with *Ulva pertusa* led to dose-dependent overexpression of IL-4 (**H**). Data are representative of at least three independent experiments. Values are means ± SD. One-way ANOVA test followed by Bonferroni. ** *p* < 0.01 vs. Sham; *** *p* < 0.001 vs. Sham; # *p* < 0.05 vs. DNBS; ## *p* < 0.01 vs. DNBS; ### *p* < 0.001 vs. DNBS.

Nuclear NF-κB translocation was found to increase after DNBS instillation (Figure 3A, see densitometric analysis Figure 3(A1)) according to the decrease of cytosolic degradation of its inhibitor IκB-α (Figure 3B, see densitometric analysis Figure 3(B1)). The treatments with *Ulva pertusa*, in a dose-dependent manner, were able to reduce the expression levels of NF-κB (Figure 3A, and densitometric analysis Figure 3(A1)) and restore the expression of Iκb-α (Figure 3B, see densitometric analysis Figure 3(B1)) comparable to the control mice. Concordant results were obtained from the analyses of p-NF-κB (Figure 3C, and densitometric analysis Figure 3(C1)) and p-Iκb-α (Figure 3D, and densitometric analysis Figure 3(D1)).

Because of the pro-inflammatory activation in UC [38], we reported that intrarectal instillation of DNBS produces an acute inflammatory response by activating IL-5, IL-9, and IL-13 release (Figure 3E–G, respectively). Reduction in the severity of DNBS ILs release was consistently observed in *Ulva*-treated mice at both doses of 50 and 100 mg/kg. Considering that IL-4 signaling could be important for the suppression of induced colitis, we observed a lower content of IL-4 in DNBS-injected mice that has been significantly restored by *Ulva* treatments at both doses of 50 and 100 mg/kg (Figure 3H).

### 3.4. Ulva pertusa Treatments Reduced Pro-Inflammatory Mediators Release of iNOS, COX-2 and Attenuated Nitrosative Stress

**Figure 4:**
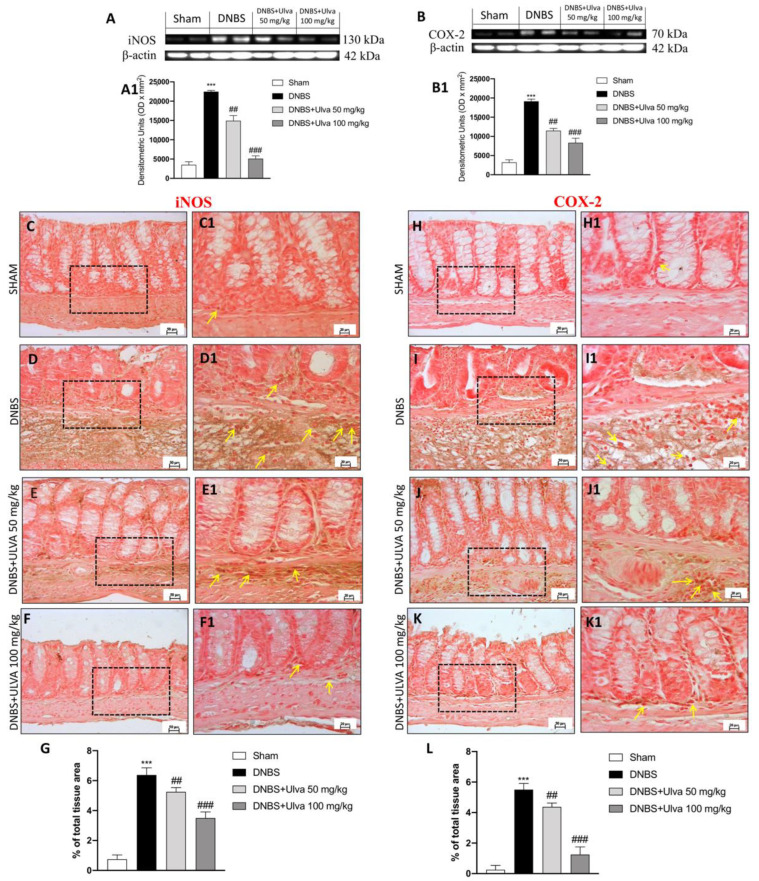
Effect of *Ulva pertusa* on pro-inflammatory mediators COX-2 and iNOS. Colon tissues collected from DNBS injected animals showed positive immunostaining for iNOS (**D**,**D1**), score (**G**), compared to the sham group (**C**), 40× magnification (**C1**), score (**G**). *Ulva pertusa* administration notably reduced such expression in a dose-related manner (**E**), 40× magnification (**E1**), (**F**), 40× magnification (**F1**), score (**G**). Western blot analysis confirmed immunohistochemical results (**A**), densitometric analysis (**A1**). Tissues from animals injected with DNBS showed positive immunostaining for COX-2 (**I**,**I1**), score (**L**) compared to the sham group (**H**), 40× magnification (**H1**), score (**L**). Administration of *Ulva* 50 mg/kg reduced this expression (**J**), 40× magnification (**J1**), score (**L**), although less effectively than *Ulva* 100 mg/kg (**K**), 40× magnification (**K1**), score (**L**). Western blot analysis of COX-2 levels confirmed the data (**B**), densitometric analysis (**B1**). Yellow arrows indicate the positive staining for iNOS and COX-2. Data are representative of at least three independent experiments. Values are means ± SD. One-way ANOVA test followed by Bonferroni. *** *p* < 0.001 vs. Sham; ## *p* < 0.01 vs. DNBS; ### *p* < 0.001 vs. DNBS.

Macroscopic damage to the colonic environment is well reported to be associated with a high expression of COX-2 and iNOS expression according to the activation of the nitrosative stress [39]. We observed that there was a strong increase of both inflammatory mediators’ expression in colon tissue of DNBS-injected mice compared to the control group (Figure 4A,B, respectively, see densitometric analysis Figure 4(A1,B1), while downregulation of iNOS and COX-2 expression was observed after *Ulva* treatments at the doses of 50 and 100 mg/kg. The present data were confirmed by detecting iNOS and COX-2 markers in colon tissue. A basal colonocytes positivity for iNOS and COX-2 markers was found in control mice (Figure 4(C1,H1), respectively, for iNOS and COX-2) despite a high increase of positive signal detected in DNBS-induced colitis mice (Figure 4(D1,I1), respectively, for iNOS and COX-2 (% of iNOS signal Figure 4G, % of COX-2 signal Figure 4L). However, a lower number of positive colonocytes for iNOS and COX-2 markers were observed in *Ulva*-treated mice in a dose-dependent manner (doses of 50 and 100 mg/kg) (Figure 4(E1,F1,J1,K1), respectively, for iNOS and COX-2).

**Figure 5:**
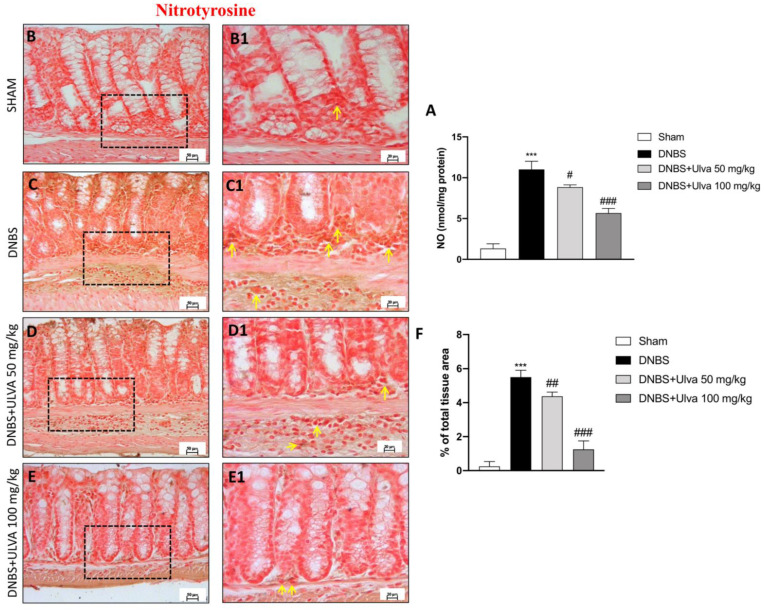
Effect of *Ulva pertusa* administration on Nitrotyrosine and NO levels. Positive Nitrotyrosine immunostaining was found in colon tissues collected from vehicle-treated mice (**C**,**C1**), score (**F**), compared to the sham-treated mice (**B**), 40× magnification (**B1**), score (**F**). *Ulva* 50 mg/kg treatment, but even more *Ulva* 100 mg/kg administration reduced this staining (**D**), 40× magnification (**D1**), (**E**), 40× magnification (**E1**), score (**F**). Concordant results were obtained from the NO assay (**A**). Yellow arrows indicate the positive staining for Nitrotyrosine. Data are representative of at least three independent experiments. Values are means ± SD. One-way ANOVA test followed by Bonferroni. *** *p* < 0.001 vs. Sham; # *p* < 0.05 vs. DNBS; ## *p* < 0.01 vs. DNBS; ### *p* < 0.001 vs. DNBS.

Since the production of NO is extensively characterizing intestinal diseases, nitrosative stress induced by NO overproduction was evaluated. Overproduction of NO was induced by DNBS intrarectal installation when compared to the control mice (Figure 5A), while *Ulva* treatments showed significant attenuation of NO production at both doses of 50 and 100 mg/kg (Figure 5A). Moreover, to determine the release of peroxynitrite and/or other nitrogen derivatives produced during colitis, nitrotyrosine, a specific marker of nitrosative stress, was also measured. Control mice colon tissue did not stain for nitrotyrosine (Figure 5(B1), contrarily, higher positive staining for nitrotyrosine was detected in DNBS-injected mice (Figure 5(C1). *Ulva*-treated mice at 50 mg/kg and more effectively at 100 mg/kg showed significantly less positive staining for nitrotyrosine staining (Figure 5(D1,E1), % of Nitrotyrosine signal Figure 5F).

### 3.5. Ulva Treatment Blockade Apoptosis Pathway Exacerbated after DNBS-Injection

**Figure 6:**
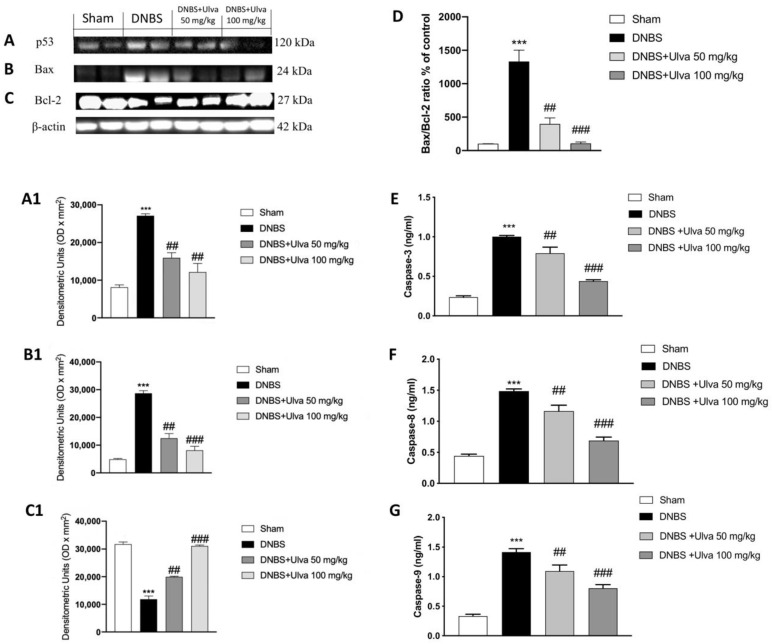
*Ulva pertusa* administration modulated apoptosis after DNBS-induced colitis. DNBS-injected mice displayed increased expression of p-53, Bax, and caspase-3, -8, -9 and diminished levels of Bcl-2, compared to the Sham group (**A**–**G** and densitometric analyses **A1**–**C1**). *Ulva pertusa* administration reduced the expression of pro-apoptotic proteins while increasing Bcl-2 levels (**A**–**G** and densitometric analyses **A1**–**C1**). Bax/Bcl-2 ratio confirmed these data (**D**). Data are representative of at least three independent experiments. Values are means ± SD. One-way ANOVA test followed by Bonferroni. One-way ANOVA test. *** *p* < 0.001 vs. Sham; ## *p* < 0.01 vs. DNBS; ### *p* < 0.001 vs. DNBS.

DNBS significantly induced cell death by increasing pro-apoptotic proteins including p53 and Bax [40]. The administration of *Ulva* substantially alleviated the apoptosis process activation; however, this effect was observed when the mice were treated with *Ulva*, particularly at the higher dose of 100 mg/kg (Figure 6A,B, see densitometric analysis Figure 6(A1,B1), respectively). We further measured the levels of anti-apoptotic player Bcl-2 and we observed an opposite trend in DNBS-induced mice such as a decreased expression level, a decrease that was restored by *Ulva* treatments at both doses of 50 mg/kg and 100 mg/kg. These results were also confirmed by Bax/Bcl-2 ratio (Figure 6D).

After administering *Ulva* to block the apoptosis, decreased expression levels of caspase 3, caspase 8, and caspase-9 were analyzed. DNBS intrarectal installation increased the levels of all pro-apoptotic caspases (Figure 6E–G). However, the effects of *Ulva* abolished the apoptotic activated process induced by DNBS-induced colitis.

### 3.6. Oxidative Stress Attenuation by Ulva in DNBS-Induced Colitis by Nrf2/SIRT1 Pathway Modulation

**Figure 7:**
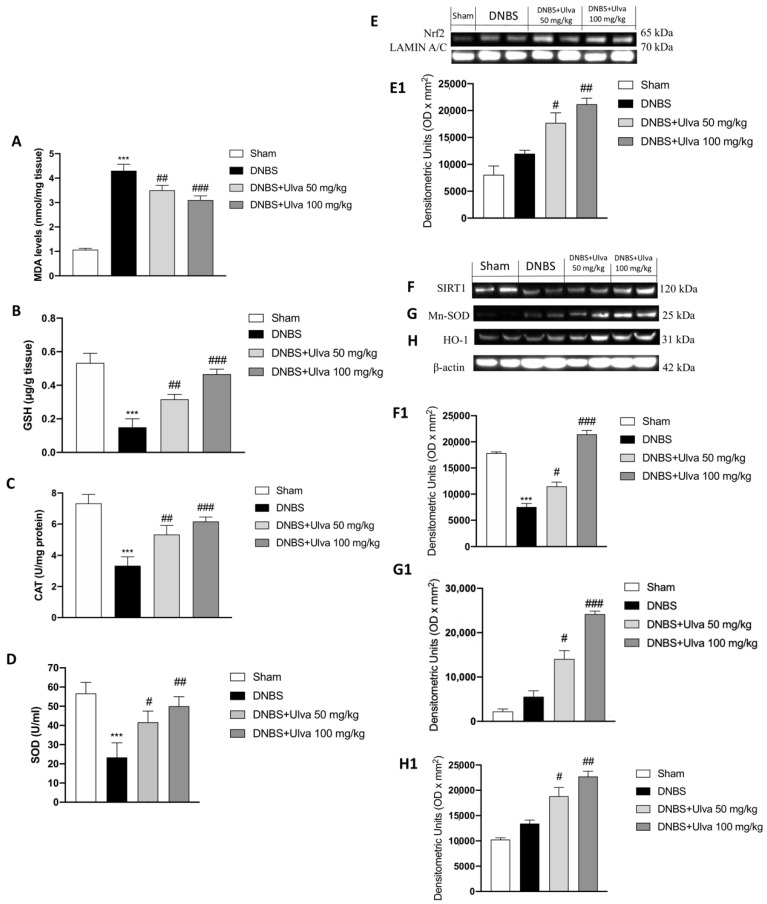
Effect of *Ulva pertusa* on antioxidant system and SIRT1/Nrf2 pathway. The administration of *Ulva pertusa* at the dose of 50 mg/kg, but more powerfully at the dose of 100 mg/kg, was able to strengthen the antioxidant response upregulating GSH, CAT, and SOD levels (**B**–**D**) and decreasing MDA levels (**A**). Furthermore, *Ulva pertusa* administration increased the expression of the nuclear factor Nrf2 (**E**), densitometric analysis (**E1**), and upregulated the enzymes MnSOD (**G**), densitometric analysis (**G1**) and HO-1 (**H**), densitometric analysis (**H1**). In addition, treatment with *Ulva*, at both doses, significantly restored SIRT1 levels which had previously been reduced by DNBS-induced colitis (**F**), densitometric analysis (**F1**). Data are representative of at least three independent experiments. Values are means ± SD. One-way ANOVA test followed by Bonferroni. One-way ANOVA test. *** *p* < 0.001 vs. Sham; # *p* < 0.05 vs. DNBS; ## *p* < 0.01 vs. DNBS; ### *p* < 0.001 vs. DNBS.

The antioxidant defense system is responsible for the removal of free radicals directly associated with inflammation of colon tissue in UC, thus offering protection against oxidative damage [41]. The increased level of MDA reflected the severity of oxidative stress induced by DNBS intrarectal instillation in mice when compared to the control. Here, *Ulva* treatments displayed great efficiency to decrease MDA levels (Figure 7A). These changes further suggest evaluating antioxidant players including GSH, CAT, and SOD. At 5 days after induction of colitis, the levels of antioxidant defenses were significantly decreased (Figure 7B–D, respectively); however, treatment with *Ulva* (50 and 100 mg/kg) potentiated their activity as reported by the increase in colon tissue of GSH, CAT, and SOD contents compared to DNBS mice (Figure 7B–D, respectively). This evidence suggests investigating mechanism-driven antioxidant activity, so our attention was focused on the Nrf2/SIRT1 pathway. Here, we observed that the treatment with *Ulva* at the dose of 50 mg/kg, even more effectively at the dose of 100 mg/kg, enhanced the antioxidant defenses by promoting Nrf2 translocation into the nucleus (Figure 7E, see densitometric analysis Figure 7(E1)) and obstacle the decrease of SIRT1 expression level, a class III NAD+-dependent deacetylase (Figure 7F, see densitometric analysis Figure 7(F1)), which had been dysregulated by DNBS-induced colitis. Moreover, expression levels of antioxidant enzymes such as Mn-SOD and HO-1 were increased following *Ulva* treatments at both doses of 50 and 100 mg/kg (Figure 7G,H, see densitometric analysis Figure 7(G1,H1)).

## 4. Discussion

The discovery of new molecular targets and unconventional care is an important goal for research and management of the intestinal disease. Concerning this, compounds of natural origin can show comparatively fewer side effects as compared to conventional methods. Previously, Wang and colleagues reported the beneficial effects of Selenized *Ulva pertusa* polysaccharides in a mice model of IBD [11]. Such findings elucidated the capability of Ulvan to promote tight junctions’ expressions and inhibit white blood cell infiltration, thus suggesting this natural compound as a potential alternative supplement for reducing intestinal inflammation in IBD. However, this green alga possesses several biological properties not yet fully explored.

Consequently, the present study aimed to investigate the therapeutical potential of *Ulva pertusa* extract, focusing on its mechanism of action as an anti-inflammatory and antioxidant mediator in an in vivo model of DNBS-induced colitis.

Nrf2, a stress-responsive transcription factor, seems to be a promising candidate for the prevention of both UC and CD [42]. Moreover, its combined role with SIRT1, a regulator that possesses physiological functions including cell proliferation, DNA repair, oxidative stress, and cell death potentiates its abilities [43]. In this framework, modulators of the SIRT1/Nrf2/NF-κB pathway could exert a protective effect against IBD consequences. Literature data provided a valuable overview of the NF-κB/Nrf2/SIRT1 signaling target as a new strategy to counteract colitis, highlighting its pivotal role in IBDs and emphasizing the inhibition of NF-κB translocation and the modulation of Nrf2/SIRT1 signal through supplements or natural compounds.

Accordingly, we found that the SIRT1/Nrf2 signaling plays the main role in attenuating oxidative stress, thanks to its crosstalk with the NF-κB pathway. In fact, we observed that DNBS-induced colitis affects the antioxidant response by SIRT1 depletion, as a result of slight binding activity of the nuclear transcriptional factor Nrf2 and reduced cytosolic expressions of antioxidant enzymes such as HO-1 and Mn-SOD. *Ulva pertusa* treatments inhibited DNBS-induced downregulation of SIRT1, by upregulating Nrf2 levels and potentiating HO-1 and Mn-SOD activity, suggesting the promotion of the antioxidant defense pathway. Moreover, despite the oxidative stress induced by DNBS, *Ulva pertusa* showed a significant improvement in the MDA, SOD, CAT, and GSH activities.

The induction of colitis in mice produced a significant decrease in body weight and intestinal disruption contributed to morphologic changes including massive infiltrative cells, edema, and necrosis. However, the treatment with *Ulva pertusa* at the two highest doses restored tissue architecture, reducing edema and polymorphonuclear neutrophilic infiltration, demonstrating a good capability to avoid body weight loss as well as histological recovery following DNBS intrarectal administration.

In IBD, adaptive mechanisms elicit an immune response, which worsens the inflammatory condition. Indeed, in the colon tissue, mast cells were found in mucosae and connective tissue, generally grouped within the epithelial layers. Our data confirmed a marked activation of the mast cells in DNBS-administered mice denoting an inflamed colon. Differently, *Ulva pertusa* daily treatment showed positive outcomes on immune cell infiltration, suggesting a good immunomodulatory capacity of *Ulva pertusa* to protect the colon from UC inflammation.

Mast cells cause inflammatory disorders by generating the products of arachidonic acid metabolism and release of preformed mediators. It has been reported an extensive exchange between mast cells and the activation of NF-κB; this interplay stimulates inflammatory cells to migrate to the inflammatory foci [44].

In accordance, we found that, after induction of colitis by DNBS, the signaling cascade through transcription factor NF-κB activation was markedly increased, as well as the level of pro-inflammatory cytokines such as IL-5, IL-9, and IL-13. *Ulva pertusa* treatment, by mediating the interplay between mast cells and NF-κB signaling, reduced these inflammatory players’ release, thus revealing powerful anti-inflammatory properties. In addition, the induction of colitis in mice by DNBS is followed by the downregulation of the expression of anti-inflammatory IL-4. The results of our study indicate that *Ulva pertusa* treatment potentiates the anti-inflammatory role of IL-4 by increasing its content. 

NF-κB activation contributes to the development and maintenance of gut inflammation. More specifically, NF-κB was found to be activated in mucosal cells of IBD patients, thus raising the assumption that its pharmacological inhibition represents a step forward in the treatment of intestinal inflammatory disorders [45]. Here, our results suggested how *Ulva pertusa* treatments exerted beneficial properties by reducing also pro-inflammatory mediators such as COX-2 and iNOS and modulating the NF-κB pathway in colitis mice.

Recent findings [46,47] emphasized the role of nitrosative stress as a determining factor in the progression and exacerbation of UC because of NO potent pro-inflammatory involvement. In this perspective, Nitrotyrosine as well as NO expression were found increased in DNBS-induced colitis mice, while *Ulva* treatments, in a dose-dependent manner, provided significant efficiency to counteract NO production and consequently nitrosative stress activation.

Several apoptosis-related protein expressions have been correlated via activating the NF-κB pathway in colonic cells of UC mice [48]. Here, the contents of p53, Bax/Bcl-2, caspase-3, caspase-8, and caspase-9 apoptosis-related proteins were investigated. When DNBS was intrarectally injected, the p53, Bax, and pro-apoptotic caspases were significantly increased in colon tissue, indicating that colitis leads to colonic cell apoptosis. We found that *Ulva* administration had an anti-apoptotic effect on UC via inhibiting the apoptosis pathway and reversing the cell death process into maintaining homeostasis of intestinal epithelial cells.

## 5. Conclusions

It can be concluded that several benefits of *Ulva pertusa* extract have been reported by demonstrating, for the first time, its powerful action to modulate the SIRT1/Nrf2/NF-κB signaling.

In light of these noteworthy biological properties, future evaluations are necessary to further investigate *Ulva Pertusa* activity, and in particular its immunomodulatory effect. Meanwhile, considering these new insights, *Ulva pertusa* could represent a promising natural support in the pharmacological strategy for IBD, improving the well-being of the gastrointestinal tract and patients’ quality of life.

Nevertheless, the limitations of preclinical models in the translational reproduction of human disorders should be considered.

In this perspective, well-designed clinical studies are needed to prove *Ulva pertusa’s* possible usefulness as dietary supplements.

This step is essential for further understanding the pharmacological potential of *Ulva pertusa* in order to focus its use on the treatment of IBD patients, thus preventing the onset of inflammatory pathology and improving their state of health.

## Figures and Tables

**Table 1:** Experimental groups and procedure of the study.

Experimental Groups	Experimental Procedure	N
Group 1: Sham + vehicle	Vehicle solution (saline) was administered by oral gavage for 4 days	10
Group 2: Sham + *Ulva pertusa* 10 mg/kg	*Ulva pertusa* extract 10 mg/kg was administered by oral gavage for 4 days	10
Group 3: Sham + *Ulva pertusa* 50 mg/kg	*Ulva pertusa* extract 50 mg/kg was administered by oral gavage for 4 days	10
Group 4: Sham + *Ulva pertusa* 100 mg/kg	*Ulva pertusa* extract 100 mg/kg was administered by oral gavage for 4 days	10
Group 5: DNBS + vehicle	Group of mice subjected to DNBS-colitis induction and then administered with vehicle solution (saline) by oral gavage every 24 h, starting from 3 h after the DNBS instillation	10
Group 6: DNBS + *Ulva pertusa* 10 mg/kg	Group of mice subjected to DNBS-colitis induction and then administered with *Ulva pertusa* extract 10 mg/kg by oral gavage every 24 h, starting from 3 h after the DNBS instillation	10
Group 7: DNBS + *Ulva pertusa* 50 mg/kg	Group of mice subjected to DNBS-colitis induction and then administered with *Ulva pertusa* extract 50 mg/kg by oral gavage every 24 h, starting from 3 h after the DNBS instillation	10
Group 8: DNBS + *Ulva pertusa* 100 mg/kg	Group of mice subjected to DNBS-colitis induction and then administered with *Ulva pertusa* extract 100 mg/kg by oral gavage every 24 h, starting from 3 h after the DNBS instillation	10

## Data Availability

All the results were included in this study and available to the corresponding author’s address.

## Supplementary Materials

The following supporting information can be downloaded at: https://www.mdpi.com/article/10.3390/jcm11154301/s1, Table S1: Composition of the macronutrients present in the *Ulva pertusa* extract. Table S2: Carbohydrate quali-quantitative composition of *Ulva pertusa* extract. Table S3: Protein quali-quantitative composition of *Ulva pertusa* extract. Table S4: Lipid quali-quantitative composition of *Ulva pertusa* extract.
