# Supplementary material for: Ulva pertusa, a Marine Green Alga, Attenuates DNBS-Induced Colitis Damage via NF-κB/Nrf2/SIRT1 Signaling Pathways
Source: J Clin Med. 2022 Jul 25;11(15):4301. doi: 10.3390/jcm11154301 (PMC9331369; doi:10.3390/jcm11154301)
Supplement: Supplementary file 1 [file jcm-11-04301-s001.zip › jcm-1804477-supplementary.pdf]

Table S1. Composition of the macronutrients present in the *Ulva Pertusa* extract

	CARBOHYDRATE	PROTEIN	LIPID	ASH
Ulva Pertusa extract Composition (100 g dry weight)	50%	25%	5%	20%

Table S2. Carbohydrate quali-quantitative composition of *Ulva Pertusa* extract

CARBOHYDRATE	*Ulva Pertusa* extract Composition (100 g dry weight)
Rhamnose	14.0%
Xylose	6.5%
Glucose	4.0%
Mannose	1.0%
Arabinose	0.5%
Galactose	0.5%
Uronic Acids	23.2%
Sulfate	17.5%
Nitrogen	1.0%

Table S3. Protein quali-quantitative composition of *Ulva Pertusa* extract

Amino acid	*Ulva Pertusa* extract Composition (g/100 g dry weight)
Aspartic acid	3.10
Serine	1.67
Glutamic acid	2.33
Glycine	1.60
Histidine	0.30
Arginine	1.40
Threonine	1.40

Alanine	2.55
Proline	0.99
Tyrosine	0.66
Valine	1.6
Lysine	1.05
Isoleucine	0.91
Leucine	1.77
Phenylalanine	1.35

Table S4. Lipid quali-quantitative composition of *Ulva Pertusa* extract

**LIPID**	*Ulva Pertusa* extract Composition (100 g dry weight)
Saturated	
14:0	2.91%
16:0	22.3%
18:0	0.5%
Unsaturated	
16:1	3.23%
18:1	10.14%
18:2 n - 6	2.11%
18:3 n - 3	12.5%
16:4 n - 3	10.9%
18:4 n - 3	15.51%
20:5 n - 6	1.46%
22:6 n - 3	1.11%
